# Crimean Congo hemorrhagic fever among the one-humped camel (*Camelus dromedaries*) in Central Sudan

**DOI:** 10.1186/s12985-017-0816-3

**Published:** 2017-08-03

**Authors:** Hajer M. Suliman, Ibrahim A. Adam, Shamseldin I. Saeed, Sanaa A. Abdelaziz, Eltahir M. Haroun, Imadeldin E. Aradaib

**Affiliations:** 1 Molecular Biology Laboratory (MBL), Department of Clinical Medicine, Faculty of Veterinary Medicine, University of Khartoum, P.O. Box 32, Khartoum North, Sudan; 2 Department of Microbiology, Faculty of Veterinary Medicine, University of Khartoum, Khartoum North, Sudan; 3 Scientific Research Directorate, Al-Mughtaribeen University, Khartoum, Sudan

**Keywords:** Epidemiology, Survey, Camels; CCHF, ELISA, Sudan

## Abstract

**Background:**

Crimean-Congo hemorrhagic fever (CCHF) is a tick-borne viral zoonotic disease caused by Crimean-Congo hemorrhagic fever virus (CCHFV), a member of the genus *Nairovirus* in the family *Bunyaviridae*. CCHF is typically asymptomatic in animals but can be highly fatal in humans approaching case fatality rate of approximately 30%. In the present investigation, a cross sectional study was conducted to determine the prevalence of CCHF and to identify the potential risk factors associated with CCHFV seropositivity among the one-humped camel (*Camelus dromedaries*) in Central Sudan.

**Methods:**

A total of 361 camels selected randomly from six localities were employed in the study. Sera sampled were tested for the presence of CCHFV-specific immunoglobulin G (IgG) antibodies using enzyme-linked immunosorbent assay (ELISA).

**Results:**

CCHFV seropositivity was recorded in 77 out of 361 animals accounting for a prevalence rate of 21.3%. Age (OR = 3.6, CI = 1.72–7.79, *p*-value = 0.026); locality (OR = 5.85, CI = 1.81–18.83, p- value = 0.003), tick number (OR = 4.6, CI = 1.37–9.81, *P*-value 0.04); tick control (OR = 2.2, CI, 1.11–4.35, *P*-value = 0.023) and breed (OR = 6.60, CI = 2.38–18.36, *P*-value = 0.001) were recorded as potential risk factors for contracting CCHF.

**Conclusions:**

The prevalence of CCHF is significantly high among camels in Khartoum State, Sudan. Age, breed, locality and tick control are considered as potential risk factors for contracting CCHF. This study would be expected to reduce the impact on the livelihood of pastoral communities and ultimately avoid disease spread in human.

## Background

Crimean Congo hemorrhagic fever virus (CCHFV) is member of the *Nairovirus* genus in the family *Bunyaviridae*. CCHF is the most widespread tick-borne viral infection of humans, occurring across a vast area from western China through southern Asia and the Middle East to southeastern Europe and throughout most of Africa. CCHFV affects both humans and a variety of animal species and hence the virus is of public health importance. CCHFV is transmitted to humans by tick bites, handling of ticks, exposure to blood or tissues of viremic livestock, or direct contact with blood and bodily fluids of infected patients [1]. Humans involved in occupations such as, slaughter house workers, shepherds, health care workers, and veterinarians are at higher risk of CCHFV [2]. Ticks of the genus *Hyalomma* are considered to be the most important vector in the transmission and the epidemiology of the disease. *Hyalomma dromedarii* is the most frequently observed tick vector associated with the disease in the one humped camel and the virus has also been isolated from ticks of other genera including, *Rhipicephalus, Boophilus, Dermacentor, Haemaphysalis, and Ixodes* spp. [3]. Despite the detection of CCHFV-specific antibodies in the dromedary camels, isolation of the virus from this animal species is yet to be reported.

Currently, little is known about the prevalence of CCHF in Sudan and no information is available with regard to the potential risk factors associated with the disease among the one-humped camel. Indirect serologic evidence of CCHFV infection was recorded in camels exported from Sudan to Egypt [4]. However, CCHF has never been reported among camels in Khartoum state, the capital of Sudan. The first outbreak of CCHF in Sudan was reported among health care workers in Alfulah rural hospital, Western Kordufan, 2008. Two virus strains designated as Al-fulah 3 and 4 were identified as etiologic agents of the nosocomial outbreak [5]. Subsequently, we reported on another outbreak in Donkup village, Abyei District, Western Kordufan, 2009 [6]. Despite the fact that the Alfulah and Abyei virus strains belong to group III genetic lineage of CCHFV, they are genetically distinct from each other and were identified as unique strains of CCHFV. A nosocomial transmission of CCHF to an attending physician in Elobied, North Kordufan was also reported as a result of medical referral of an infected patient from Lagawa district of Western to Northern Kordufan, Sudan. The identified virus strains including, Lagawa, Abyei and Alfulah are considered to be responsible for the recent emergence of CCHF in Sudan [5–7].

Recently, we reported on a CCHF prevalence rate of 7% during an epidemiological survey of the disease among cattle from North Kordufan state of Sudan [1]. Subsequently, another cross sectional study was carried out among cattle in East Darfur state, Sudan, which showed a high prevalence rate of 19.14% [8]. The detection of specific-CCHFV circulating antibodies in camels suggests that this animal species may harbor the virus [3, 4]. It is worth mentioning that, during CCHFV infection, viremic camels can provide virus for vector transmission to human populations. Therefore, the control of hemorrhagic fever including CCHF and Rift Valley fever virus (RVF) would be important in the Sudan given the large numbers of camels in the country, and their importance to the national economy and rural communities [9]. We believe the epidemiologic studies including implementation of improved surveillance would be necessary to prevent possible CCHF outbreaks in Sudan [10]. The objectives of the present investigation were to estimate the prevalence of CCHFV IgG antibodies and to identify potential risk factors associated with the diseases among the one humped camel (*Camelus dromedaries*) in Khartoum state, Central Sudan. We anticipate that this study would reduce the impact of the disease on rural and pastoral communities and ultimately prevent transmission of the disease in human population living in CCHF areas of endemicity.

## Methods

### Study area

**Fig. 1:**
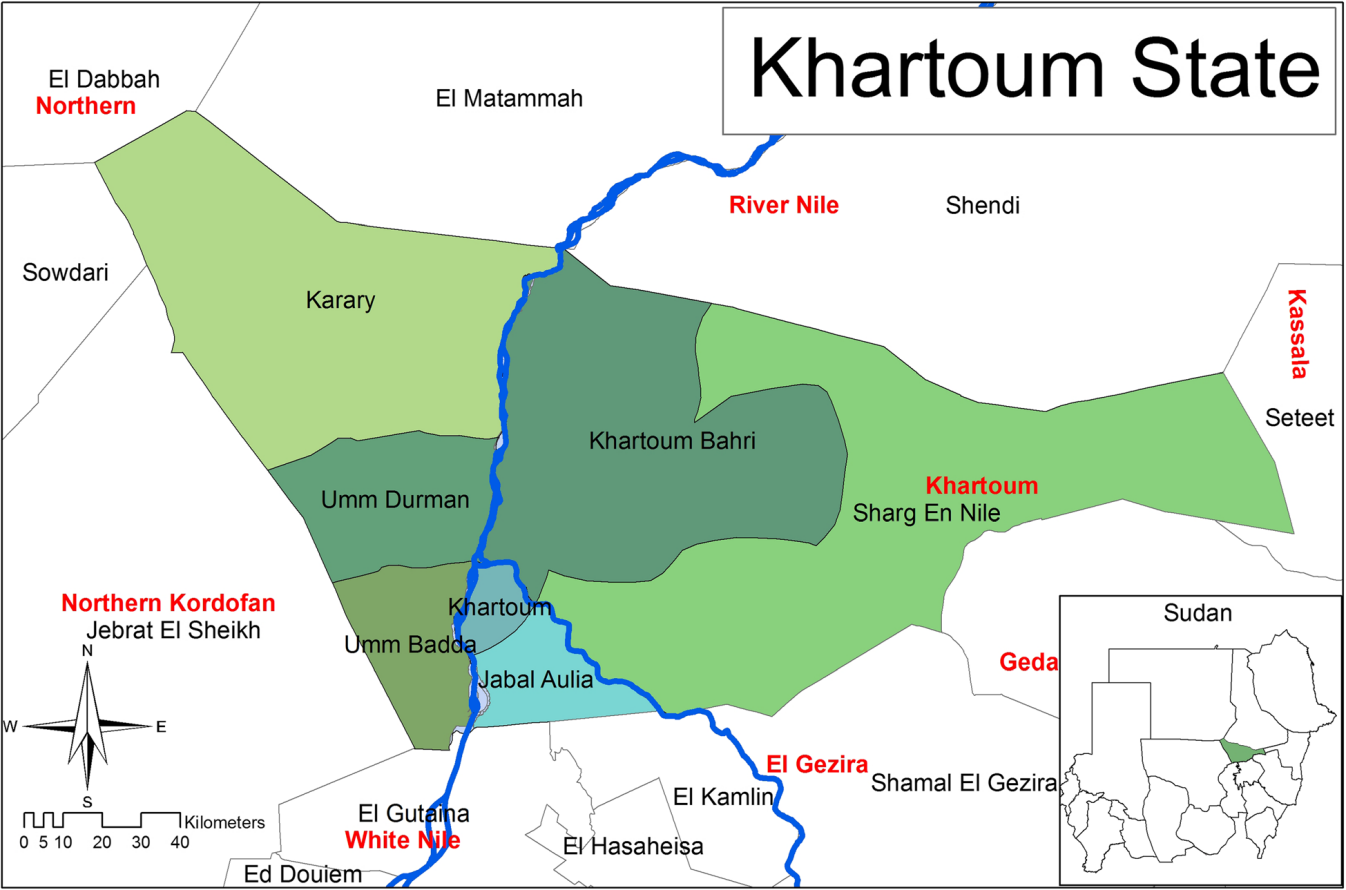
A map of the localities included in the study area of Khartoum State

The study was carried out in Khartoum State from October 2014 to March 2015. The State covers an area of approximately 20,971 km^2^. The State is located between latitudes 15° 8° - 16° 39° N and longitudes 31° 36–34° 25 E in the semi desert tropics. It is dominated by semi desert climate, which is characterized by very hot/dry summer and cold winter. The average temperature ranges from 21 °C in the winter to 47 °C in the summer. The mean annual evaporation rate is 7.7 mm/day, and the average relative humidity ranges from 21% to −38%. Khartoum State is boarded by the River Nile State in the north; Kassala States in the east, North Kordufan in the west and Elgezera State to the South. The total population of camels in the country is approximately 4.6 million. The camel population of Khartoum State is 6585 as estimated by the Sudan Ministry of Animal Resources, 2006 [9]. A map of the localities included in the study area of Khartoum State is presented in (Fig. 1).

### Study design

A cross sectional study was conducted to estimate the prevalence rate of CCHFV-specific Ig G antibodies in camels and to investigate the potential risk factors associated with the disease. A total of 361 camels were selected randomly using multistage probability sampling method. Six localities were randomly selected from all seven localities in Khartoum State, Sudan, which include, Um Badah, Omdurman, Sherq En nile, Bahri, Jabal Awlia and Karary. A simple random sampling method was applied to choose the animals from each herd [10]. All camels included in this study were aged over one year.

### Questionnaire

A pre-tested structured questionnaire with the primary objective of elucidating the multifactorial background of disease was used to survey the animal owners of all selected herds in and interactive manner. The animals included in this study were subjected to a questionnaire, which was filled out by the animal owners. The questionnaire include individual risk factor attributes including age (younger animals <2 years, older animals 2 years and above), sex (male, female), breed (Anafi, western, Bushari), body condition (emaciate, thin, fat),and management risk factor attributes including herd size. Regarding herd size, small herd size means >20, medium means 21–40 and large means > 40, grazing system (nomadic,, stationary), ticks vector (presence or absence), tick control (practiced or not), farm yard (indoor or outdoor) and the six localities included in the study.

### Ethics approval and consent to participate

Collection of blood from camels was performed by qualified veterinarians following proper physical restraint of animals to ensure both personnel and animal safety. Livestock owners were explained the study purposes before procedures and upon agreeing to participate, they provided a written consent prior to study procedures and blood collection from their animals. The study received ethical clearance from the Research Board of the Faculty of Veterinary Medicine, University of Khartoum, Sudan. The risk factor information was obtained from the animal owners through the structured questionnaire form, which permitted the use of the blood samples for diagnostic and research purposes.

### Collection of blood samples

A total of 361 blood samples were collected from the jugular vein of the selected dromedary camels in the study area of Khartoum State, Central Sudan. Serum samples were separated and were kept frozen at −20 °C until used for detection of CCHFV-specific IgG antibodies using indirect enzyme-linked immunosorbent assay (ELISA).

### Enzyme-linked immunosorbent assay (ELISA)

Indirect enzyme-linked immunosorbent assay (ELISA) was performed to screen the sera for CCHFV-specific immunoglobulin G (IgG) antibodies basically as described by [8]. ELISA was performed in 96-well immunoassay microplates (Nunc, Roskilde, Denmark) and optimal working dilutions of reagents were determined by chessboard titration. Unless stated otherwise, 100 μl (μl) test volumes were used, incubations were performed for 1 h at 37 °C. The plates were washed three times with PBS containing I% Tween 20 (Merck, Darmstadt, Germany) (PBST), wells were post-coated with 200, μl of PBS containing 2% bovine serum albumin (Calbiochem, La Jolla, USA), and the diluents for reagents was PBS containing 10% Skimmed milk (Amba, Denmark). Briefly, the plates were coated with sucrose-acetone extracted CCHFV antigen and incubated overnight at 4 °C. The source of the antigen used was cell lysate from CCHFV Nigeria strain IbAr 10,200. The antigen used in this study was obtained from the Center for Disease Control and Prevention, Atlanta, USA). The plates were washed, and aliquots of test sera (positive and negative controls) were added in separate wells at a dilution of 1:100. After a 1-h incubation, the plates were washed, and rabbit anti-camel IgG conjugated with horse radish peroxidase (HRP) was added to the plate at a dilution of 1:1000 and incubated for 1 h. The plates were then washed and the substrate, 2,2- azino-bis (3-ethylbenthiazoline-6-sulfonic acid, (Kirkegaard and Perry Laboratories) was added. CCHFV-infected camel serum sample was incorporated in each ELISA plate as positive control to estimate the higher limit of the sensitivity. Negative control sera were used to estimate the lower level of specificity of the ELISA assay and were obtained from CCHFV-free animals and from camel infected with Rift Valley fever virus (RVFV), a hemorrhagic fever virus of the same family (*Bunyaviridae*). The results were read by using ELISA reader set at 405 nm. A presumptive diagnosis was made when IgG antibody titer in the test sample had optical density (O.D) value of <0.20.

### Statistical analyses

Entry of the data in the computer was made possible using statistical package for social sciences (SPSS) software package for window (version 21.0) and double checked before analyses. Logistic regression analyses were performed using the seropostivity to CCHFV Ig G as dependent variable and the risk factors as independent variables. Odd ratios and 95% confidence interval (C.I) were calculated and *P* value <0.05 was considered statistically significant.

## Results

This study showed that 77 out of 361 camel serum samples were found to be positive for CCHFV-specific-IgG antibodies, accounting for an overall prevalence rate of a 21.3% among camels in Khartoum State, Central Sudan. Univariate analysis using Chi-square test was conducted for the association between the potential risk factors and CCHFV seropositivity and *p*-value (*p* < 0.25) was initially considered significant. The results of the univariate analysis showed that the independent variables including, age, breed, tick number, body condition, farm yard, tick control and locality were statistically significant (Table 1). The risk factors that were significant in the univariable model were re-entered into a final multivariate model using logistic regression analysis. In the final models, a variable with a *p*-value <0.05 was considered statistically significant.

**Table 1:** Summary of analysis for risk factors of (CCHF) among Camels in Khartoum state, Sudan (*n* = 361 camels) by using chi-square test

Risk factors	Animals tested	Animals affected (%)	df	*χ*2	*p*-value
Locality			5	44.74	0.001
East Nile	60	5 (8.3%)			
Bahry	60	8 (13.3%)			
Omdurman	60	23 (38.3%)			
Ombadda	60	3 (5%)			
Jabal Awlia	60	12 (20%)			
Karary	61	26 (42.6%)			
Age			1	5.64	0.01
Small	73	23 (31.5%)			
Old	288	54 (18.8%)			
Sex			1	0.70	0.48
female	309	65 (21%)			
male	52	12 (23%)			
Breed			2	17.39	0.001
Western	128	13 (10.4%)			
Anafi	58	21 (36.2%)			
Bushari	237	43 (24.2%)			
Body condition			2	3.58	0.16
Emaciation	3	0 (0%)			
Thin	136	23 (16.9%)			
Fat	222	54 (24.3)			
Farm yard			1	7.03	0.008
In door	172	47 (23.3%)			
Out door	189	30 (15.9%)			
Grazing system			1	0.50	0.47
Stationary	241	54 (22.4%)			
Nomadic	120	23 (19.2%)			
Herd size (camels/ herd)			2	2.30	0.31
Small <20	72	16 (22.2%)			
Medium = 21–40	52	15 (28.8%)			
Large >40	237	46 (19.4%)			
Ticks present			2	1043	0.005
No	26	11 (42.3%)			
Small	125	31 (24.8%)			
large	210	35 (16.7%)			
Ticks control			1	8.54	0.003
No	283	51 (18%)			
Yes	78	26 (33.3%)			

Age (OR = 3.6, CI = 1.72–7.79, *p*-value = 0.026); locality (OR = 5.85, CI = 1.81–18.83, p- value = 0.003), tick number (OR = 4.6, CI = 1.37–9.81, *P*-value 0.04); tick control (OR = 2.2, CI, 1.11–4.35, *P*-value = 0.023) and breed (OR = 6.60, CI = 2.38–18.36, *P*-value = 0.001) were recorded as potential risk factors for contracting CCHF. The results are summarized in (Table 2). In contrast, there was no significant association between CCHFV seropositive camels and other individual or management risk factors included in the study.

**Table 2:** Multivariate analysis, using logistic regression model, for significant association (*p* < 0.05) of risk factors and CCHF seropositivity among camels in Khartoum State, Sudan

Risk factors		OR	95%C I	*P*-Value
Age	small	Ref		
	old	3.6	1.72–7.79	0.026
Locality	Ombadda	Ref		
	Karary	5.85	1.81–18.83	0.003
Ticks number	NO	Ref		
	large	4.6	1.37–9.81	0.04
Ticks control	Yes	Ref		
	No	2.20	1.118–4.35	0.023
Breed	Western	Ref		
	Bushari	6.6	2.38–18.36	0.009

## Discussion

Sporadic cases and frequent outbreaks of CCHF have recently been frequently reported worldwide including the Sudan. The risks these cases pose for medical staff in resource poor health care facilities necessitate the importance of improved surveillance system for this important emerging viral pathogen [5–7, 9–17]. It is well documented that CCHFV does not induce overt clinical hemorrhagic disease in host animals. The only evidence that the virus is circulating in a given geographic focus is the occurrence of the disease in human populations. The one-humped camel may harbor the virus and develop CCHFV-specific antibodies [18]. The routine surveillance for CCHF and associated vector transmission in sentinel camel herds would be the backbone of the epidemiological study of CCHF in a particular area of endemicity. In the presence of a competent tick vector, the risk of infection to local residents will depend upon the population density of the tick, the prevalence of infected ticks, and the frequency of bites [19]. Tick bite transmission or exposure to blood or tissues of infected livestock is more likely to occur during rainy season in the Sudan, when roads become very muddy and transport between villages or cities becomes extremely difficult, if not impossible [6]. Under these environmental conditions, the local residents typically live in close contact with their camels within their home villages, increasing the risk of acquiring infection from infected livestock or by bite of infected tick [5]. It is worth mentioning that during civil war, as is the case in Darfur and South Kordufan regions of western Sudan, displaced people living in camps are likely to be forcibly exposed to the local environment. Under these circumstances, the refugees will be in close proximity to their animals and CCHF-infected ticks, which will lead to a higher risk of infection. Should CCHF cases be admitted to medical facilities or rural hospitals with resource poor settings, nosocomial chains of transmission would be expected to occur particularly if effective infection control practices are lacking [5–7, 11, 15].

Camels were selected in this study as CCHFV infection appears to occur most frequently in larger mammals, which are the preferred hosts of adult tick vector, *Hyalomma dromedaries* [18, 20]. In spite of the presence of the *Hyalomma* tick vector, Sudan was thought to be CCHF-free and infection with CCHFV has never been reported in the country until 2008 [5]. This study expands on the existing data indicating that CCHF, known to be endemic in Africa, is broadly distributed within Sudan. It is worth mentioning that Sudan shares boarders with different countries including Egypt, Ethiopia, Eriteria, Kenya, Uganda, Chad, Libya, Central African Republic, Democratic Republic of Congo, and the State of South Sudan. It would be interesting to trace the movement of the virus between these African countries and to identify the virus genetic lineages circulating in the African continent. Our results indicated that, the overall prevalence of CCHFV IgG-specific antibodies was 21.3%. The CCHFV IgG-specific anti-bodies recorded in this study showed evidence of prior exposure of camels to CCHF in Khartoum State of Central Sudan. The high prevalence rate (21.3%) indicates significant circulation of CCHFV among camel in Central Sudan and that human population in the region may have also been infected with CCHFV. The CCHFV-specific antibodies detected among camels in this study indicate natural infection as there is no vaccination program for this disease. In addition, all camels included in this study aged over one year. Therefore, it is assumed that maternal antibodies no longer persisted and that antibody indicated direct exposure to CCHFV [1, 21, 22].

In the present study, there was significant difference between the CHFV seropositivity rate and the age of the animal, stressing the intense and long lasting exposure of the camel population to CCHFV. We believe that the association of CCHFV infection rate and age is probably attributed to frequent exposure of older camel calves to infected tick in the pasture. In addition, the study revealed significant association between tick-infestation and CCHFV seropositivity. It is well documented that CCHF is a tick-borne zoonotic disease and that heavily tick-infested camels are likely to become CCHFV positive by bites of infected ticks [23–25]. It should also be noted that treatment of camels with insecticides should be applied monthly to prevent tick infestation. Small vertebrates such as hares and hedgehogs, which are infested by immature ticks, may be particularly important as amplifying hosts [18]. The ground-based birds such as ostrich can amplify the virus. The role of migratory birds in CCHF transmission between distant geographic areas should also be taken into consideration [26, 27]. Furthermore, there was a significant difference between CCHF seropositivity and localities assigned in this study. The highest seropositivity was recorded among camels in the locality of Karrari, which is attributed to the dry climate and variations in temperatures that provide favorable environmental condition for the tick vector. The risk assessment studies indicated that there was no significant difference between CCHFV seropositivity and the rest of the individual or management risk factors included in the study. Both sexes are equally affected by CCHFV as gender has no significant difference for CCHFV infection among male and females. The residents should also be educated about the risks of the disease, and prevention of the infection through tick control program. In this regard, effective animal husbandry and management system should be applied to control tick infestations in domestic livestock populations. Veterinarians and health care providers should be aware of the disease and be prepared for a possible outbreak in the region. Physicians and medical health workers in Khartoum State should consider CCHF in their efforts to diagnose the disease in patients with clinical presentations compatible with those of CCHF [7, 28–30].

## Conclusions

In conclusion, the prevalence of CCHF is significantly high among camels in Khartoum State, Sudan. Age, breed, locality and tick control are considered as potential risk factors for contracting CCHF. This study would be expected to reduce the impact on the livelihood of pastoral communities and ultimately avoid a disease spread in human during a possible CCHF outbreak. Further molecular characterization studies such as, complete viral genome sequencing and subsequent phylogeny would be necessary to determine the genetic lineages of CCHFV strains circulating in infected tick vectors in Sudan.

## Abbreviations

CCHF: Crimean Cong Hemorrhagic fever
CCHFV: Crimean Congo hemorrhagic fever virus
CI: Confidence interval
ELISA: Enzyme-linked immunosorbent assay
HRP: Horse radish peroxidase
Ig G: Immunoglobulin G
OR: Odd ratio
RVFV: Rift Valley fever virus
